# Depression, Help-Seeking and Self-Recognition of Depression among Dominican, Ecuadorian and Colombian Immigrant Primary Care Patients in the Northeastern United States

**DOI:** 10.3390/ijerph120910450

**Published:** 2015-08-27

**Authors:** Susan Caplan, Steven Buyske

**Affiliations:** 1 School of Nursing, Rutgers, The State University of New Jersey, 228 Ackerson Hall, 180 University Avenue Newark, NJ 07102, USA; 2 Department of Statistics and Biostatistics, Hill Center, Rutgers, The State University of New Jersey, 110 Frelinghuysen Rd. Piscataway, NY 08854, USA; E-Mail: buyske@stat.rutgers.edu

**Keywords:** depression, help-seeking, stigma, immigrants, self-recognition, self-perceived need

## Abstract

Latinos, the largest minority group in the United States, experience mental health disparities, which include decreased access to care, lower quality of care and diminished treatment engagement. The purpose of this cross-sectional study of 177 Latino immigrants in primary care is to identify demographic factors, attitudes and beliefs, such as stigma, perceived stress, and ethnic identity that are associated with depression, help-seeking and self-recognition of depression. Results indicated that 45 participants (25%) had depression by Patient Health Questionnaire (PHQ-9) criteria. Factors most likely to be associated with depression were: poverty; difficulty in functioning; greater somatic symptoms, perceived stress and stigma; number of chronic illnesses; and poor or fair self-rated mental health. Fifty-four people endorsed help-seeking. Factors associated with help-seeking were: female gender, difficulty in functioning, greater somatic symptoms, severity of depression, having someone else tell you that you have an emotional problem, and poor or fair self-rated mental health. Factors most likely to be associated with self-recognition were the same, but also included greater perceived stress. This manuscript contributes to the literature by examining attitudinal factors that may be associated with depression, help-seeking and self-recognition among subethnic groups of Latinos that are underrepresented in research studies.

## 1. Background and Significance

Mental illnesses and substance abuse disorders are the leading causes of disability world-wide [1]. Among mental illnesses, depression is the second leading cause of disability in high-income countries, such as the United States, [2] and is predicted to become the leading cause of disability worldwide, affecting from 11% to 15% of the global population during their lifetimes [3]. In a recent United States national survey, one in five people reported current depressive symptoms [4]; however, the majority of people with depression were not receiving care [5,6].

The absence of treatment for depression is a particularly severe problem for people of Hispanic origin (Latinos). Latinos are the largest minority group in the United States, representing 17% of the population [7]. By 2060, it is predicted that Latinos will make up 31% of the United States population, or approximately one in three residents [8]. Although past-year mental illness and depression rates for Latinos are similar to or slightly less than those for the population as a whole, 15.3 *vs.* 19 (any mental illness) and 6.9% *vs.* 7% (for depression) respectively, this differs by ethnic background, nativity and generational status [9,10]. It is important to recognize that, in most research studies, Latinos have been lumped together, but, in reality, cultural between-group differences may be far greater than commonalities. For example, Puerto Ricans have higher rates of mental illness than non-Hispanic Whites and other Latino ethnic groups and greater rates of treatment [9,11].

### 1.1. Risk Factors for Depression

Many Latinos have demographic characteristics that increase their vulnerability to depression; unemployment, poverty and low educational attainment [12,13]. Among primary care populations, refugees, and monolingual Spanish-speaking populations, there is a high prevalence of affective disorders, estimated at 20%–45% due to the above-mentioned factors associated with depression, in addition to chronic illness and exposure to violence or chronic stress [14,15,16]. In addition, for Latino immigrants, experiences of loss of previously existing social support systems and changes in cultural values and social roles are stressors that have been associated with depression, along with perceived discrimination and neighborhood safety [15,17].

### 1.2. Mental Health Care Disparities

Latinos suffer from large health disparities when compared to non-Hispanic Whites [10,11,18]. Only half of Latino immigrants with severe mental health disorders receive any type of mental health treatment [10].The rates of mental health service use from 2008 to 2012 for any mental illness were 27.3% for Hispanics compared to 46.3% for non-Hispanic Whites. These mental health disparities also include decreased access to care, and less guideline-consistent care in the form of antidepressant usage, appropriate referrals for mental health care and duration of treatment [4,18,19]. Disparities in access to and quality of mental health treatment are due, in large part, to lack of health insurance, language barriers and poverty [10,11,18]. However, when taking into account these factors, Latino mental health care utilization is still half that of non-Hispanics Whites [10].

### 1.3. Provider-Level Barriers to Treatment of Depression

Depression is far more frequently diagnosed and treated in the primary care setting than in specialty mental health care [5,20]; in 2012, 59% of adults in the United States with Major Depressive Disorder (MDD) received care in the primary care setting [5]. When Latinos do seek mental health care services, it is most often in the primary care sector [21]. Therefore, primary care providers are in a unique position to provide screening for depression and ensure access to care. Although the United States Preventive Services Task Force (USPSTF) has recommended screening of all patients for depression in the primary care setting when systems are in place to provide appropriate treatment and follow-up [22], most patients do not receive screening [23]. Instead, primary care providers rely on their own clinical judgment, which is much less effective in detecting depression than the use of formal screening instruments [24]. Access to treatment is hindered by lack of recognition in primary care in as many as 30% to 60% of patients [25,26]. Primary care providers’ recognition of depression is increased the more often a patient is seen [27]. However, many Latinos and individuals with limited English proficiency do not have a usual source of health care [28].

Twenty-four percent of Spanish speakers are linguistically isolated, meaning that no adult household members speak English well or at all [7]. This affects provider recognition of depression. The most important predictor of help-seeking behavior is the provider’s ability to speak the patient’s language [29]. Only 11% of the behavioral health workforce is Hispanic [30]. Thus, the lack of providers who are bilingual and/or bicultural contributes to inadequate recognition.

### 1.4. Individual Barriers and Facilitators of Treatment

Patient-level factors strongly influence decisions to seek help for mental health problems, particularly in undiagnosed conditions when cultural factors and level of acculturation becomes particularly salient [31]. One particular cultural value among Latinos, *Familismo,* or the belief in strong family connections and unity, is believed to have a major influence upon help-seeking, both positive and negative [32]. The alternative theory of help-seeking hypothesizes that due to the cultural value of *Familismo* people with depression may seek out family members or faith-based leaders rather than professional help [33]. Moreover, the value of *Familismo* may influence help-seeking either due to stigma or reluctance to disclose personal problems to outsiders or fear of burdening one’s family or guilt about being depressed [34,35]. Other beliefs, such as the importance of self-reliance and fear that therapy will bring up too many bad feelings, can create a barrier to help-seeking [33,36,37,38,39,40].

Perceived need for mental health care is often operationalized to reflect previous experiences with help-seeking or among people already receiving treatment, the voluntary nature of treatment [41]. Factors contributing to perceived need include history of chronic physical conditions, history of depression or anxiety and severity of symptoms and prior experience with mental health treatment among multi-ethnic participants [41,42]. Barriers to self-perceived need are minority and Limited English Proficiency status, as well as stigma [43,44,45].

### 1.5. Self-Recognition of Depression

Self-recognition combines the concepts of perceived need with that of self-rated health, which has been defined as the intersection of biology and culture since it relies upon lay meanings of symptoms [46]. Self-rated health has consistently been a predictor of mortality, primarily because it encompasses more than a biomedical definition of health, and includes functional abilities, social relationships and spirituality [46]. In one study of Puerto Ricans, depressive symptoms strongly predicted self-rated health [47]. However, research has also indicated Latinos are less likely than non-Hispanic Whites to accurately self-assess their mental health status and to seek help when their self-rated mental health was poor [48] or they may label their illness as “stress” [38]. Thus, self-recognition of depression is a precursor but not a prerequisite of help-seeking. To date, only a few studies have addressed factors related to self-recognition of mental illness among Latinos and some of these studies were completed more than ten years ago [49,50,51,52]. Results of these studies indicated that between 21% and 62% of participants were able to self-recognize depression or another mental health disorder. However, the upper end of this range represents Internet users who were likely to be more highly educated and of higher socio-economic status than the population in the proposed research. The most salient factors enabling self-recognition were severity of illness, suicidality, history of self or family/friend mental health treatment, somatic symptoms and poor self-perceived mental health [49,50,52]. Those who believed in a biomedical explanation of illness were also more likely to acknowledge a mental health problem [49,51]. Research in the area of symptom presentation and other subjective perceptions that contribute to self-recognition is limited as is research related to help-seeking for depression among Latino immigrants in the primary care setting.

The purpose of this study is to identify demographic factors, attitudes and beliefs such as stigma, perceived stress and ethnic identity that are associated with depression, and help-seeking among Latino immigrants in primary care. An exploratory analysis will be conducted to see how these factors might be associated with self-recognition of depression.

Based on the literature we hypothesize that:

(1) Greater somatic symptoms, poverty, female gender, negative attitudes and perceived stress will be associated with depression.
(2) Functional status, severity of depression, higher level of acculturation, female gender, less negative attitudes and perceived stress will be associated with help-seeking.
(3) Functional status, severity of depression, higher level of acculturation, female gender, less negative attitudes, having someone else state that you have a problem and perceived stress will be associated with self-recognition.

To address the burden of untreated behavioral health problems, understanding of factors associated with depression and help-seeking behaviors is critical. The addition of assessments about attitudes about depression treatment as well as immigrant stressors adds to the literature which has addressed economic and linguistic barriers to help-seeking but frequently has not informed us of the subjective facilitators or barriers to help-seeking and self-recognition, particularly for Latino immigrants. In this manuscript we contribute to the literature by studying Spanish-speaking residents of the United States, who are predominantly Dominican, Ecuadorian and Colombian, a population of Latino immigrants that is growing rapidly but has received less attention in the past [53].

## 2. Methods

### 2.1. Participant Characteristics

Participants were patients at a private family practice in Corona, Queens, New York, which has an urban population of 99,000. Approximately two-thirds of the population in Corona is Hispanic; of these 62% are foreign born [7]. One-quarter of the Hispanic residents live below the poverty level. The site was selected because of it had a predominantly Latino immigrant patient population and the particular demographic mix of the population of predominantly Dominican, Ecuadorian and Colombian immigrants.

### 2.2. Procedures and Recruitment

This research was a cross-sectional mixed-method design, using questionnaires and semi-structured interview questions. The sample for this study consisted of a convenience sample of self-identified Latino immigrants recruited from the waiting room of the practice upon registering for their appointments. Eligible participants endorsed that they were Latino, age 18 or over and were born either outside of the United States or in Puerto Rico. Over the four-month recruitment period, 177 participants were enrolled.

The first author and Principal Investigator accompanied eligible individuals who expressed interest in participating to a private room in the clinic for a formal consent and interview, where further details and full disclosure of the study’s purpose was provided. The first author is fluent in Spanish and a native English speaker with extensive research experience with Latino populations, particularly among Caribbean Latinos in the Northeastern United States and the Dominican Republic. The study received Institutional Review Board (IRB) approval from a university-based IRB. Subjects were interviewed in Spanish or English according to their preference, and the instruments were read aloud to participants in their language of choice. In conjunction with the structured interview, an in-depth informal interview technique was employed using spontaneous, informal conversation and further elicitation for clarification [54]. Participants had the option of expanding upon the Likert Scale responses to the instruments described below such as the Abbreviated Version of the Hispanic Stress Inventory for Immigrants (HSI-I) and were prompted to give examples or illustrations of their responses. Their explanatory comments were transcribed verbatim. A detailed description of this data is described in Caplan *et al.*, 2010 [55].

### 2.3. Measures

All measures used in this study had pre-existing validated Spanish translations that had utilized the back translation method [56]. Two independent native Spanish speakers translated instructions and consent forms. The investigator pretested or reviewed the measures for comprehension and usage among monolingual Spanish speakers from Mexico, Puerto Rico, the Dominican Republic, Ecuador and Colombia, which were countries most likely to be represented in the sample.

### 2.4. Dependent Variables.

#### 2.4.1. Depression

The Patient Health Questionnaire (PHQ) depression module (PHQ-9) [57] is a 9-item scale with a sensitivity of 88% and a specificity of 88% for major depression at a score of greater than or equal to 10 [58]. In our sample α = 0.90.

#### 2.4.2. Help-Seeking

History of help-seeking was ascertained by the item, “Have you ever gone to a doctor or other health care professional for emotional problems, mental health problems or problems with alcohol or drugs?”.

#### 2.4.3. Self-Recognition of Depression

Self- Recognition of depression required a PHQ score greater than 5 and either a yes response to (a) below or a “fair” or “poor” rating to (b) below:

- In the past month have you ever had personal, emotional, behavioral, or mental or alcohol or drug problems severe enough that you felt you needed help [49]?
- Self-rated mental health: How would you rate your overall mental health—excellent, good, fair or poor [52]?

### 2.5. Independent Variables

#### Clinical/Exposure Variables

*Self-Rated Mental Health and Self-Rated Physical Health* were ascertained by the items, “How would you rate your overall mental (physical) health—excellent, good, fair or poor?”

*Being told by someone else they had a mental health problem* was ascertained by the question: “During the past year, did another person (family member, friend, neighbor, minister, priest or other) think that you had a problem with nervios, emotional problems, or use of drugs or alcohol?”

*Total Somatic Symptoms*. The Somatic Symptoms scale of the PHQ contains 15 items about physical complaints rated from 0, 1, and 2, corresponding to “not at all,” “bothered a little” and “bothered a lot.” In our sample α = 0.85.

*Functional Status* was assessed by the single question on the PHQ: “How difficult have these problems made it for you to do you work, take care of things at home or get along with other people?” It is scored on a four-point scale ranging from “not at all difficult” to “extremely difficult.”

*Acculturative Stress* was measured by the HSI-I [59]. The abbreviated version is a 17-item questionnaire designed to assess stressors specific to the Latino immigrant experience, such as immigration, discrimination and family and cultural issues. Internal consistencies were acceptable across all subscales, ranging from .68 to .83. Convergent validity of the abbreviated HSI-I revised was supported with positive relations through self-report measures of depression, anxiety and anger mood levels [59]. We eliminated items on the subscale pertaining to parental stressors since they were not relevant to the majority of our sample, who had grown children. In our sample α = 0.89.

*Perceived Stigma* contained 3 items reflecting beliefs that one would be stigmatized by employers, friends and family for seeking treatment for depression [51]. In our sample α = 0.71.

*Demographic variables* included age, gender, educational level and income level.

## 3. Analysis

Analyses were conducted using the R statistical environment (R Core Team, 2015) [60]. Among the full sample, the individual scales representing Total Somatic Symptoms, the HSI-I and Perceived Stigma were analyzed through univariate descriptive statistics to describe the means, range and standard deviations of the scores on these items.

Bivariate statistics were used to examine the association between presence or absence of depression, presence or absence of help-seeking and presence or absence of self-recognition and differences by the categorical variables of Functional Status, presence or absence of chronic illness, the number of chronic illnesses, income, gender, Self-Rated Mental Health and Self-Rated Physical Health and being told by someone else that you had a mental health problem using a χ^2^ test or Fisher’s Exact test, depending on cell size. The continuous variables of age, Total HSI, Total Somatic Symptoms and Perceived Stigma were examined for an association between presence or absence of depression and presence or absence of help-seeking by using T-Tests or Analysis of Variance*.*

Lasso logistic regression, a form of penalized logistic regression that favors parsimonious models, was used to build statistical models of association. Different starting sets of predictor variables were used for each dependent variable, based on a significant *p* value of 0.05 in the bivariate analyses. These variables were:

**Depression**: Functional Status, presence or absence of chronic illness, Self-Rated Mental Health, Self-Rated Physical Health, Perceived Stigma, Income, Total Somatic Symptoms and Total HSI-I;

**Help-Seeking**: Functional Status, gender, Self-Rated Mental Health, Self-Rated Physical Health, being told by someone else that you had a mental health problem, Self-Recognition, Total PHQ score and Total Somatic Symptoms and;

**Self-Recognition**: Functional Status, gender, Self-Rated Physical Health, being told by someone else that you had a mental health problem, Self-Recognition, Total PHQ score, Total Somatic Symptoms and Total HSI-I.

Cross-validation was used to determine the size of the lasso penalty. As no generally accepted method exists for calculating p-values following the lasso, we fit logistic models showing only the variables with non-zero coefficients in the lasso-fit. The resulting nominal p-values are likely to be anti-conservative. The C-index was used to assess the model adequacy; it estimates the probability that, for a randomly selected case and control, the predicted risk is higher for the case [61,62]. Models with c ≥ 0.80 are considered to have good discrimination in predicting risk.

## 4. Results

### 4.1. Sample Characteristics

Sociodemographic and clinical characteristics of the sample are presented in Table 1. The largest ethnic group in this sample was of Dominican origin, followed by those of Ecuadorian and Colombian origin.

### 4.2. Clinical Characteristics

Forty five-participants (25%) had depression by Patient Health Questionnaire (PHQ-9) criteria [58]. Two other participants did not meet PHQ-9 criteria, but were currently in treatment for depression. Another 33 (19%) of participants did not meet PHQ-9 criteria, but believed that they needed help with mental health problems such as depression. The depression severity was rated as follows: 7 (16%) had mild depression, 16 (36%) had moderate depression, 14 (31%) had moderately severe depression and 8 (19%) had severe depression. Twelve (27%) were receiving treatment for depression. Of the 33 who were not receiving treatment 21 (62%) knew that they had an emotional problem that might require treatment (self-recognition), whereas 12 (38%) did not believe that they had a problem (non-self-recognition).

**Table 1:** Baseline Demographics (n = 177).

Variable	Mean (SD)
Total Perceived Stigma * (range = 3–15)	8.5 (3.8)
Total Somatic Symptoms (range = 0–28)	7.4 (5.9)
Total Hispanic Stress Inventory (range = 0–70)	17.5 (15.9)
Variable	N (%)
*Demographics*	
**Age**	
18–34	20 (11)
35–49	63 (36)
50+	94 (53)
**Gender**	
Male	48 (27)
Female	129 (73)
**Income Level**	
<$10,000	63 (36)
$10,000–$19,999	64 (36)
>/=$20,000	36 (20)
Refused or don’t know	14 (8)
**Educational level**	
8th grade or less	46 (26)
Some high school	56 (32)
GED or high school	33 (19)
Some college or technical school	22 (12)
College Grad or Grad school	20 (11)
Ethnicity	
Dominican	91 (51)
Ecuadorian	28 (16)
Colombian	25 (14)
Other Central/South Amer.	33 (19)
*Acculturation Variables*	
**Number of years in country**	
0–5 (1)	19 (11)
6–10 (2)	11 (6)
11–15 (3)	25 (14)
15–20 (4)	26 (15)
20+ (5)	95 (54)
**Language Usage**	
Spanish only	56 (32)
Mostly Spanish (some Eng.)	95 (54)
Spanish and English about the same	22 (12)
Mostly English (some Spanish)	4 (2)
English only	
**Language Preference**	
Spanish	170 (96)
English	7 (4)
*Clinical/Exposure*	
**Number of Chronic Illnesses**	
0	72 (41)
1	50 (28)
2	22 (12)
3 or more	33 (19)
**Self-rated Physical Health**	
Excellent or Good	87 (49)
Fair or Poor	90 (51)
Self-rated Mental Health	
Excellent or Good	121 (68)
Fair or Poor	56 (32)
**In the past month have you had problems that you thought you needed help with?**	
Yes	40 (23)
No	137 (77)
**History of previous help seeking**	
Yes	54 (31)
No	123 (69)
**History of Medical Provider telling you that you had a problem (when help seeking n = 54)**	
Yes	45 (83)
No	9 (17)
**History of Medical Provider telling you that you had a problem (w/o help seeking)**	
Yes	12 (6)
No	165 (94)
**History of previous treatment (of the 66 who sought help or were told by an medical provider that he/she had a problem)**	
Yes	48 (73)
No	18 (27)
**History of Family/Friend telling you that you had a problem**	
Yes	26 (15)
No	151 (85)
**Total Depression by PHQ**	45 (25)
Major Depression	32 (71)
Other Depression	13 (29)
Total Current Treatment w/Dep	12/45 (27)

* Low score = low stigma.

#### 4.2.1. Depression

Twenty-five percent of the population had a PHQ score indicative of depression. In the bivariate analysis, factors most likely to be associated with depression were: income less than $10,000 annually; somewhat to extreme difficulty in functioning (Functional Status); greater somatic symptoms; greater HSI-I score; greater perceived stigma; presence of chronic illnesses and increased number of chronic illnesses and poor or fair self-rated mental health (see Table 2). Using lasso logistic regression, the final selected model for depression consisted of the factors functional status, poor or fair self-rated mental health, and Total Somatic Symptoms, with a C-index for the model of C = 0.895. Controlling for the other terms in the model, Functional Status and Total Somatic Symptoms were significant predictors of depression.

#### 4.2.2. Help Seeking

Fifty four people (31%) answered yes to the question: “Have you ever gone to a doctor or other health care professional for emotional problems, mental health problems or problems with alcohol or drugs?” Of the fifty-four participants, 45 (83%) were told by their medical provider that he/she had a problem with emotions, alcohol or drugs. Thirty-five participants were told specifically that he/she had depression and/or depression and anxiety.

In the bivariate analysis, factors most likely to be associated with help-seeking were: female gender; somewhat to extreme difficulty in functioning; having someone else tell you that he/she thought you had a mental health or emotional problem; Total Somatic Symptoms; total PHQ-9 score; and poor or fair self-rated mental health and physical health status (see Table 3). The final logistic regression model for help-seeking consisted of female gender; having someone else tell you that he/she thought you had a mental health or emotional problem; poor or fair self-rated mental health and Total Somatic Symptoms. This model had a C-index of 0.749. Poor or fair self-rated mental health and Total Somatic Symptoms were nominally significant after controlling for the other terms (see Table 4).

#### 4.2.3. Self-Recognition

Sixty percent (32) of the participants self-recognized their depression. Factors most likely to be associated with self-recognition were: female gender; somewhat to extreme difficulty in functioning; having someone else tell you that he/she thought you had a mental health or emotional problem; Total Somatic Symptoms; HIS-I; total PHQ-9 score; and poor or fair self-rated mental health and physical health status in the bivariate analysis (see Table 3). The final logistic regression model for self-recognition consisted of having someone else tell you that he/she thought you had a mental health or emotional problem and Total PHQ, with the C-index for model of C = 0.758. Only Total PHQ showed nominal significance after controlling for the other term (see Table 4).

**Table 2:** Bivariate Associations with PHQ-9 score indicative of depression.

Characteristic	Depression Present n = 45 (25%)	Depression Absent n = 132(75%)	*p* Value
*Demographics*			
**Age**			NS (0.47)
18–49	19 (42)	64 (48)	
50+	26 (58)	68 (52)	
**Gender**			NS (0.64)
Male	11 (23)	37 (28)	
Female	34 (76)	95 (72)	
**Income Level**			0.047
<$10,000	21 (48)	42 (35)	
$10,000–$19,999	19 (43)	45 (38)	
</= $20,000	4 (9)	32 (27)	
*Clinical/Exposure*			
**Function**			<0.0001
Somewhat to Extremely Difficult	32 (71.1)	16 (14.8)	
Not at all difficult	13 (28.9)	92 (85.1)	
**Chronic Illness**			0.0014
yes	36 (80)	70 (53)	
no	9 (20)	62 (47)	
**Number of Chronic Illnesses**			0.0016
0	9 (20)	62 (47)	
1 or 2	21 (47)	51 (39)	
3 or more	15 (33)	19 (14)	
**Self-rated Physical Health**			<0.0001
Good/Excellent	11 (24.4)	76 (57.6) ‚	
Fair/Poor	34 (75.6)	56 (42.4)	
**Self-rated Mental Health**			<0.0001
Good/Excellent	16 (35.6)	105 (79.6) ‚	
Fair/Poor	29 (64.4)	27 (20.4)	

**Continuous Variables**	**Mean (SD)**	**Mean (SD)**	***p* Value**
Total Acculturative Stress (mean)	29.3 (16.1)	13.5 (13.7)	<0.0001
Perceived Stigma (range = 3–15)	9.9 (3.8)	8.0 (3.6)	0.0042
Total Somatic Symptoms (range = 0–24)	13.5 (6.0)	5.3 (4.1)	<0.0001

* Variables at *p* ≤ 0.05 were entered into the multiple regression equation.

## 5. Discussion

The demographic, psychosocial and clinical factors associated with depression are consistent with the majority of epidemiological studies of depression in the community and in primary care patients, with the exception of the lack of gender differences. Poverty [19], poor self-perceived physical health and poor self-perceived mental health [48] are all factors previously associated with depression. The presence of other chronic conditions, as well as the number of chronic conditions has been shown to increase the odds of depression [63,64,65]. Similar to the results of the current study, Nadeem *et al.* (2007) [45] found that, among Latinos, stigma is more prevalent among those who are depressed than in those without depression. Among foreign-born and less acculturated Latinos, perceived stigma may be an important barrier to quality of mental health treatment [66,67]. Stigma about depression is related to causal attributions. In this group of participants, level of perceived stigma was highest among participants who believed that depression was the result of personal transgressions, injustice or malevolent spiritual forces [55]. With the exception of perceived injustice, these causal attributions are aligned with the theory of attribution and stigma which suggests that outcomes perceived as less controllable or due to external factors elicit greater sympathy and support compared to those attributed to personal (internal) factors such as poor judgment, divine retribution, personal weakness, or sinfulness [68]. Results of this study are evidence of the theory of Link and Phalen (2001) [69] that mental illness stigma disproportionately affects already marginalized groups, contributing to further discrimination.

**Table 3:** Bivariate associations with help seeking and self-recognition.

Characteristic	Help Seeking Yes n = 54 (31%)	Help Seeking No n = 123 (69%)	Self-Recognition Yes n = 32 (60%)	Self-Recognition No n = 12 (30%)
*Demographics*				
**Gender**				
Male	9 (19) *	39 (81)	5 (42)	7 (58)
Female	45 (35)	84 (65)	27 (79)	7 (21)
**Educational Level**				
<High School	28 (28)	73 (72)	23 (79)	6 (21)
High School grad or greater	26 (34)	50 (66)	9 (53)	8 (47)
*Clinical/Exposure*				
**Functional Status**				
Somewhat to Extremely Difficult	26 (54) **	22 (46)	26 (79) *	7 (21)
Not at all Difficult	25 (22)	91 (78)	6 (46)	7 (54)
**Someone else thought you had an emotional problem**				
Yes	17 (65) **	9 (35)	12 (38) *	1 (07)
No	37 (25)	114 (75)	20 (62)	13(93)
**Self-rated Physical**				
Good/Excellent	17 (20) **	70 (80)	5 (42) *	7 (58)
Fair/Poor	37 (41)	53 (59)	27 (79)	7 (26)
**Self-rated Mental**				
Good/Excellent	23 (19) **	98 (81)	N/A	N/A
Fair/Poor	31 (55)	25 (46)		
**Continuous Variables**	Mean (SD)	Mean (SD)	Mean (SD)	Mean (SD)
**Total Hispanic Stress Inventory (mean)**	25.27 (22.0)	16.3 (13.8)	33.78(16.6) *	16.43 (14.4)
**Total Perceived Stigma** (range = 3–15)	8.53 (4.0)	8.50 (3.7)	9.94 (3.6)	9.29 (4.7)
**Total Somatic Symptoms** (range = 0–24)	10.53 (6.9) **	5.79 (4.5)	14.55 (6.0) **	9.43 (5.1)
**Total PHQ**	9.33 (7.9) **	4.11 (5.3)	16.41 (5.3) **	10.85 (4.81)
**Age**	48.46 (10.4)	51.18 (12.9)	50.78(11.4)	49.07(15.5)

* *p* < 0.05; ** *p*< 0.005.

**Table 4:** Logistic regressions.

Variable	Help Seeking			Self Recognition			Depression		
	Point Estimate	SE	*p* Value	Point Estimate	SE	*p* Value	Point Estimate	SE	*p* Value
Gender	−0.299	0.452	0.508						
Someone else telling you that you had a mental health problem	0.765	0.539	0.156	0.67	1.256	0.595			
Functional Status							−1.81	0.53	0.00058
Self-Rated Mental Health	−1.022	0.404	0.011				−0.36	0.52	0.49592
Total PHQ-9				−0.19	0.088	0.029			
Total Somatic Symptoms	−0.093	0.037	0.012				−0.19	0.05	0.00011

The association between perceived stress, depression and self-recognition of depression confirms the results of previous research [70,71]. Among primary care populations, reports of recent stress of any type are the most significant predictors of depression [72]. The content of the items indicated in the HSI-I [59], such as economic difficulties, intergenerational conflict and discrimination may create day-to-day hassles [73] and could also result in chronic stress that has been associated with depression [74]. Results of previous research support the notion that recent traumatic events are a factor in the subsequent development and severity of depression [75,76,77,78,79]. Nevertheless, the causal pathway of perceived stress and depression remains unclear from these results. As has been suggested in other studies, perceived stress may be greater in individuals with depression because of differences in coping styles and cognitions that are affected by depression [80,81].

Unlike the majority of epidemiological studies in the population, this study does not show gender differences in prevalence of depression [82,83]. There are a couple of probable theories for this. Depression in Latino men may be related to changing gender roles, but has also correlated with unemployment, financial stressors, failure to fulfill social expectations and perceived discrimination. In this population, interviewed at the height of the recession, the unemployment rate among immigrants was 31%. This theory is also given plausibility by the finding in a study of low-income Puerto Ricans that men who experience long-term unemployment were much more likely to be treated for a mental health problems than women [84]. However, results of this investigation conform to our knowledge of gender differences in help-seeking behavior [48]. In this study, women were significantly more likely to seek help for depression than men and to self-recognize their depression. Gender differences in help-seeking behavior can be explained by men’s adherence to traditional male gender roles, which reinforce the idea that help-seeking is associated with weakness and vulnerability [85]. Latino men may be particularly susceptible to fear of being stigmatized for help-seeking behavior because of the predominant ethos of *Machismo* which has been shown to be a barrier to prostate screening [86] and help-seeking for psychological problems [87]. Moreover, restrictive emotionality or a narrowed range of perceived emotions has been linked to *Machismo*, traditional male gender roles and help-seeking for psychological problems, and may be hypothesized to be a mediator of the decreased self-recognition among men found in this study.

Controlling for the psychosocial, attitudinal and demographic factors that were significant in the bivariate analysis, only functional status and somatic symptoms were associated with depression. The results of this study are similar to others, which have shown that depression has been associated with impaired functional status, increased work absenteeism and decreased quality of life [88,89]. For Latinos, this association is attenuated in some research [14,90], which may be explained by the tendency among Latinos with depression to deny work-related or functional disability regardless of severity of depression because of employment-related demands and lack of sick leave [90].

Research has indicated that, in primary care populations, those with anxiety and depressive disorders report higher numbers of medically unexplained symptoms, controlling for the symptoms of comorbid chronic medical illness [27,91]. Thus, it becomes imperative for primary care providers to have a high index of suspicion of depression in patients with medically unexplained symptoms. Nevertheless, in this study, increased somatic symptoms did not create a barrier to help-seeking or self-recognition of depression. In fact, the converse seems to be true.

A frequently touted theory is that minorities fail to self-recognize depression because of a tendency to somaticize, which obscures emotional symptoms of distress [48,92]. This may be due, in part, to lack of familiarity with mental health treatment in their countries of origin, the stigma of reporting mental illness or the patient’s beliefs about what symptoms are acceptable to report in a primary care setting [93,94]. However, this has not been substantiated and was not the case in this study [95]. Recent evidence, including the results of this study, suggests the opposite is true, that for first-generation Latinos, physical symptoms increase the likelihood of self-perceived need for care [96]. Results of our study indicated that severity of depressive symptoms predicted self-recognition, similar to the results of other research studies [47,49,50,52], in which functional status, number of medical conditions and severity of depressive symptoms predicted self-rated health.

This group of immigrants was relatively homogenous in terms of level of income, acculturation status and Spanish-language usage. Thus, it is unsurprising that acculturation variables did not have an effect on help-seeking or self-recognition of depression. This is consistent with some previous research of Caribbean Latino populations [36]. Somewhat unexpectedly, stigmatizing attitudes did not predict help-seeking or self-recognition, unlike the results of a study by Jang *et al.* (2011) [36] where a belief that “having depression would make family members disappointed,” was associated with help-seeking. In the study by Jang *et al.* [36], although family fear of disappointing family members was significant in the regression analysis, only 2.7% endorsed a fear of disappointing family members, whereas in our research that number reached 19%. Nevertheless, this study illustrates the complexities of the concept of *Familismo.* While disappointing family members due to one’s depression is clearly a concern for these participants, family members are also a conduit to help-seeking and self-recognition. In contrast to the alternative resource theory of the family as an alternative to seeking help in the medical sector [49], results of this research indicated that family members’ recognition of a problem could facilitate help-seeking. Moreover, compared to previous research [49,50,51,52], this research showed high rates of self-recognition. This could be due to the effects of the media, pharmaceutical advertising and public health campaigns related to treatment of mental illness and depression, which have increased in the intervening years since the previously cited research was conducted. Public health campaigns have been successful in increasing recognition of biomedical treatments and understanding of depression as a biomedical illness [97]. However, one area of future research would be to see how such public health campaigns have affected the attitudes of Latinos in the United States.

### Limitations

The sample consisted of an immigrant primary care population from different Latin American countries, which limits the possibility of generalizing to all Latino primary care populations in the United States. The study is further limited by a convenience sampling strategy and a variable refusal rate. The number of participants with depression in this study does not represent the prevalence of depression in the clinic, because not all patients were assessed and some patients may have self-selected into the study in order to be screened or to get help for depression. Nevertheless, 25% is within the range of results of other primary care studies of Latino immigrants and low-income primary care patients [12,14,26,98].

The refusal rate varied from approximately 75% initially to about 10% by the end of the study, when the investigator was well known to the potential participants, some of whom had already participated in the study. Due to HIPPA regulations and the need to protect patient privacy, only minimal eligibility data was obtained from potential participants in the waiting room and no other personal identifying information was obtained from non-participants that could subsequently be used to compare participants with those who refused to participate. Neither could information be obtained as to documented or undocumented status, which would influence help-seeking behavior or presence of health insurance, although the majority of patients in this population received Medicaid or Medicare.

Although the PHQ is based on DSM-IV criteria and shows high agreement with clinical diagnosis as evidenced by an overall accuracy rate of 88%, the absence of a clinical interview to verify the PHQ results obtained here may mean that some participants may have been misclassified as “depressed” or “not depressed.” Since this is a primary care population, it would be assumed that there is some overlap of somatic and psychiatric comorbidities, so one cannot definitely discern if impairment is due to chronic illness or symptoms of depression. Lastly, the cross-sectional design of this analysis precludes identification of the causal directions of the variables under study.

## 6. Conclusions and Implications for Future Research

Disparities in access to and quality of mental health care continue to be a significant issue for Latinos. Thus, knowledge about factors related to depression, help-seeking and self-recognition is a necessary first step to begin to reduce these disparities. With the passage of the Affordable Care Act (ACA), there is a greater impetus to address the mental health needs of Latinos, particularly in the primary care setting [99,100]. However, primary care providers frequently do not identify cases of depression [25,26,101]. In addition, Latino immigrants may not volunteer their own feelings of depression to medical providers. Results of this research point to a way out of this particular dilemma. The high rates of self-recognition in this study may have been amplified by the addition of the concept of self-rated mental health. Thus, one simple, fast and potentially effective screening method would be to add in an open-ended question on self-rated health: “How would you rate your health? Responses could range from 1 to 5: 1—Excellent (Excelente), 2—Very Good (Muy Buena), 3—Good, (Buena), 4—Fair (Regular), 5—Poor (Mala) with fair of poor requiring further screening.

The high rates of depression among Latinos in primary care and the demographic and clinical characteristics that contribute to these high rates of depression point to the need for effective culturally adapted depression prevention strategies. Cultural adaptation includes such modifications to conventional psychoeducational interventions as: explicitly addressing culture, using client’s preferred language, collaborating with significant others, and including culturally relevant discussion [102]. Results of this study provide a roadmap for these modifications. Collaborating with significant others and encouraging communication with the patient who is depressed could enhance help-seeking and facilitate treatment engagement. Given the association between HIS-I and self-recognition, immigrant stress might be an important factor to incorporate into a culturally relevant discussion, particularly since patients with depression may not perceive themselves to be depressed, and may talk about stressful life events, rather than symptoms of depression. Cardemil, Kim, Pinedo and Miller (2005) [103] created a culturally appropriate depression prevention program for low-income Latina mothers that incorporated group discussions of immigrant stressors, such as experiences of prejudice, adapting to a new environment, and having children grow up in a foreign culture. Incorporation of this specific cultural content validated participants’ experiences and created trust.

In addition to the more obvious effects of language barriers among Latinos, quality of care and treatment retention in primary care is greatly influenced by the depth of patient-provider communication and patient satisfaction with communication [104]. This is often unsatisfactory because of the decreased likelihood that primary care providers will talk to minority patients about mental health care [105]. Communication can be enhanced by eliciting the client’s beliefs about the etiology of their illness, and their prediction of its severity and the consequences of illness. Acknowledging the participant’s beliefs of how immigrant stressors may contribute to depression is a way of honoring the participant’s explanatory model. Eliciting and validating the explanatory model of illness is perhaps the most critical component of a culturally adapted depression intervention [106].

Lastly, given the preponderance of the evidence linking depression to poverty, chronic illness and stress, it would be worthwhile to explore interventions that address these more upstream factors associated with depression. Interventional research designed to ameliorate these social determinants of depression might be the most effective means to prevent depression among Latinos.

Declarations: Data and research materials can be accessed by contacting the author.
